# Temperature-Derived Purification of Gold Nano-Bipyramids for Colorimetric Detection of Tannic Acid

**DOI:** 10.1021/acsanm.3c01593

**Published:** 2023-06-27

**Authors:** Yuxiang Xue, Xinyao Ma, Xue Feng, Sam Roberts, Guangyu Zhu, Yi Huang, Xianfeng Fan, Jun Fan, Xianfeng Chen

**Affiliations:** † School of Engineering, Institute for Bioengineering, University of Edinburgh, The King’s Buildings, EH9 3JL Edinburgh, U.K.; ‡ Department of Materials Science and Engineering, City University of Hong Kong, 83 Tat Chee Ave, 00000 Kowloon Tong, Hong Kong, SAR, P. R. China; § Department of Chemistry, City University of Hong Kong, 83 Tat Chee Ave, 00000 Kowloon Tong, Hong Kong, SAR, P. R. China; ∥ School of Engineering, Institute for Materials Processing, University of Edinburgh, The King’s Buildings, EH9 3JL Edinburgh, U.K.

**Keywords:** Gold nanobipyramids, gold nanorods, gold nanoparticles, purification, sensing

## Abstract

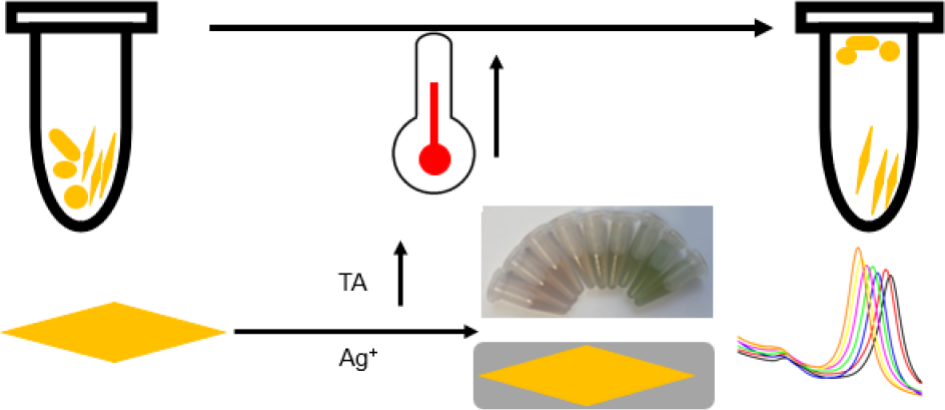

Gold nanostructures have attracted broad attention. Among various nanostructures, gold nanobipyramids have shown great potential in sensing, biomedicine, environmental protection, chemical catalysis, and optics due to their unique physical and optical properties and ease of chemical functionalization. Compared with other plasmonic nanostructures, gold nanobipyramids possess narrow optical resonances, stronger plasmonic local field enhancement, and size- and shape-dependent surface plasmon resonance. However, the synthesis and purification of homogeneous gold nanobipyramids are very challenging. The gold nanobipyramids synthesized via the commonly used seed-mediated growth method have low yields and are often coproduced with spherical nanoparticles. In this study, we reported a temperature-derived purification method for the isolation of gold bipyramids. In the presence of salt, by altering the temperature of the solution, large gold bipyramids can be separated from small spherical nanoparticles. As a result, a yield of as high as 97% gold nanobipyramids can be achieved through a single round of purification, and correspondingly, the ratio between the longitudinal surface plasmon resonance (LSPR) and transverse SPR intensity significantly increases to as high as 6.7. The purified gold nanobipyramids can be used as a colorimetric probe in the detection of tannic acid with a detection limit of 0.86 μM and a linear detection range from 1.25 to 37.5 μM.

## 1 Introduction

With the fast development of nanotechnology, gold nanostructures have attracted great attention in research.^1−4^ Compared with bulk gold, gold nanostructures exhibit distinct properties and functionality. One of their most prominent features is their unique and tunable optical properties. When gold nanostructures are exposed to light, there is a strong interaction between the photon and the conduction electrons on the surface of the gold nanostructures, leading to a collective oscillation, termed surface plasmon resonance (SPR). The optical properties of gold nanostructures are highly related to their size and shape. For example, with the increase of the radius, spherical gold nanoparticles (AuNPs) exhibit stronger and broader absorbance peak with redshifts; by altering the aspect ratio of gold nanorods (AuNRs), the SPR peak can be adjusted from the visible region to infrared region.^5,6^ Similarly, gold nanobipyramids (AuBPs) possess a wide range of adjustable SPR peaks.^7^ More attractively, due to the sharp end-tips, AuBPs have stronger local electric field enhancements and better optical properties.^5,8^ For these reasons, AuBPs are considered to be promising plasmonic nanoprobes in sensing. In order to achieve the lab to real-world use of AuBPs, it is essential to obtain monodisperse AuBPs via an economical and convenient method.

The seed-mediated growth method is the most common and reliable one to synthesize AuBPs. However, the yield of AuBPs is subjected to criticism, as some studies reported it to be only approximately 30% while the rest of the product was composed of 60% of pseudo-AuNPs and 10% of AuNRs.^9^ To solve the problem of low yield of AuBPs, several purification methods have been developed.^10^ For example, purification through depletion-induced flocculation has shown great progress in isolation AuBPs.^11−13^ Studies reported that this purification approach could be carried out at ambient temperature.^11,13^ However, the result could not be replicated in our lab, and after a systematic study, we proved that the purification process is highly temperature-sensitive, which means that the same purification approach carried out in different regions or seasons may lead to conflicting results and far inferior separation of AuBPs from other structures.

Herein we report a temperature-derived purification method to purify AuBPs and study the mechanism with coarse-grained (CG) molecular dynamics (MD) simulations. To the best of our knowledge, this is the first report of controlling the temperature instead of the salt concentrations and types for the highly efficient purification of AuBPs. Upon the addition of electrolytes, the structure of cetyl trimethyl ammonium bromide (CTAB) micelles change from spherical to wormlike. By alteration of the temperature, the length of CTAB micelles can be adjusted. At an optimal temperature, large AuBPs precipitate, while small AuNPs remain stable in solutions. Through a single round of purification, the purity of AuBPs can be significantly improved from 47% to over 93%. Due to the distinctive optical properties of nanosized AuBPs, they can be used as a colorimetric probe to detect tannic acid (TA), achieving a detection at 0.86 μM and a linear range of 1.25 to 37.5 μM.

## 2 Materials and Methods

### 2.1 Materials

Tetrachloroauric acid trihydrate (HAuCl_4_·3H_2_O, 99%), cetyltrimethylammonium bromide (CTAB, 98%), hexadecyltrimethylammonium chloride (CTAC, 25% in water), sodium chloride (NaCl, 99%), sodium nitrate (NaNO_3_, 99%), sodium carbonate (Na_2_CO_3_ 99.5%), silver nitrate (AgNO_3_, 99%), sodium borohydride (NaBH_4_, 98%), hydrogen chloride (HCl, 37%), sodium citrate dihydrate, sodium hydroxide, sodium salicylate (NaSal), tannic acid, glutamine (Gln), urea, D-glucose (Glu), and glycolic acid (GA) were all purchased from Sigma-Aldrich. Sodium acetate (NaOAc) was purchased from Honeywell Research Chemicals. L-Ascorbic acid was purchased from Alfa Aesar. D(-)-Quinic acid (QA) and CaCl_2_ were ordered from Fisher Scientific. All chemicals were used as received without any further purification. Ultrapure water (resistivity 18.20 MΩ cm at 25 °C) was used in all experiments.

### 2.2 Synthesis of AuBPs

**Scheme 1:**
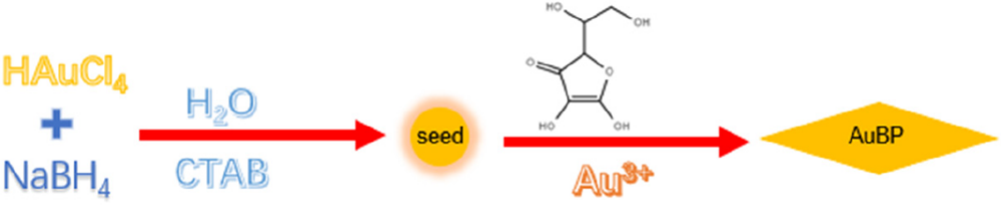
Schematic of the Preparation of AuBPs

AuBPs were synthesized by a two-step seed-mediated growth method, by using CTAB as the template and surfactant (Scheme 1). In the first step, the Au seeds were prepared according to a previous study with minor modification.^14^ Briefly, the seed solution was prepared by adding 100 μL of 20 mM HAuCl_4_ into 8 mL of 66 mM CTAC solution. Then, 100 μL of 50 mM/50 mM NaBH_4_-NaOH mixture solution were added to the seed solution under moderate stirring followed by addition of 100 μL of 0.1 M sodium citrate dihydrate. After 5 min, the seed solution was heated to 80 °C for 1 h. The growth solution was prepared by adding 5 mL of 20 mM HAuCl_4_, 2.5 mL of 10 mM AgNO_3_, and 4 mL of 1 M HCl into 200 mL of 0.1 M CTAB solution. Then 1.6 mL of 100 mM ascorbic acid was added to the mixture followed by moderate stirring for 30 s. After a colorless solution was formed, 1.5 mL of seeds solution was added to the growth solution followed by vigorous shaking for 30 s. Finally, the solution was left undisturbed overnight at 30 °C for the growth of AuBPs.

### 2.3 Purification of AuBPs

After synthesis, 200 mL of AuBPs solution was divided into four 50 mL centrifuge tubes followed by centrifugation at 7600 rpm for 20 min (Eppendorf Centrifuge 5430). Subsequently, the supernatant was removed, and the precipitation was washed twice by adding 50 mL of water to each tube and then centrifugation with the same speed and time. Finally, the washed precipitate was resuspended in 50 mL of 5 mM CTAB solution. For purification, 4 mL of the above solution was placed in a centrifuge tube followed by adding 6 mL of 4 M different salt solutions (NaCl or NaNO_3_). Then, the test tubes were placed in an incubator at different temperatures without disturbance for 12 h. Next, the supernatant mainly composed of small gold nanoparticles was removed, and the pellet mainly composed of purified AuBPs was redispersed in 5 mL of 10 mM CTAB and sonicated by sonication. Finally, the AuBPs were washed twice with 10 mL of 10 mM CTAC by centrifugation at room temperature to remove excess salts and redispersed in 10 mL of a 10 mM CTAC solution. In this work, all centrifugation procedures were carried out at room temperature.

### 2.4 Colorimetric Detection of Tannic Acid

The purified AuBPs were diluted with 10 mM CTAC solution until the optical density (O.D.) of the LSPR peak (800 nm) reached around 1.5. To detect TA, 900 μL of AuBPs solution was placed in an Eppendorf tube followed by addition of 30 μL of 0.1 M AgNO_3_. Subsequently, 20 μL of 1 M Na_2_CO_3_ was added to adjust the pH to around 6.6. For detection, 200 μL of the samples with different concentrations of TA was individually added to the probe solution containing AuBPs. Finally, the mixture was placed in a thermomixer at 60 °C for 70 min with moderate shaking.

### 2.5 Characterization

Transmission electron microscopy (TEM) was carried out on a JEOL JEM1400 microscope with an accelerating voltage of 80 kV. The ultraviolet–visible-near-infrared (UV–vis-NIR) spectra were recorded by a NanoDrop 2000c spectrophotometer, using a cuvette of 10 mm path length.

### 2.6 MD Simulations

The coarse-grained molecular dynamics (CG MD) simulations were performed using GROMACS 2020 package.^15^ The systems were solvated in standard MARTINI CG water molecules, including sodium and the corresponding anions, to achieve a neutral target ion concentration. Two types of anions were considered, including chloride ions or salicylate ions, to elucidate the effects of anion type on the shape properties of CTAB micelles.^16,17^ To avoid freezing, 10% of the total water particles were modeled using the antifreeze CG water provided in the MARTINI force field database. The initial dimension size of each system is 20 × 20 × 20 nm^3^. The surfactant concentration was set as 0.1 M, while two different NaSal concentrations, 0.05 and 0.1 M, were adopted to evaluate the effects of salt concentration on CTAB micelles features. Each system was modeled with MARTINI force field.^17^ The mapping strategy of atomistic CTAB and NaSal into CG beads and the corresponding force field parameters were obtained from previous work, which have been proven to capture the shape transition characters of CTAB micelles.^18−20^ Each system was first subject to energy minimization and then a relaxation process lasting for 10 ns, followed by a production run of 2 μs. The time step for integrating Newton’s equations of motion was 25 fs. The systems were simulated at NPT ensembles. The temperature was maintained at target value using a V-rescale thermostat while the pressure was kept at 1 bar using Parrinello-Rhaman barostat.^21,22^ Snapshots were generated and rendered by Visual Molecular Dynamics (VMD) software.^23^

## 3 Results and Discussion

### 3.1 Synthesis and Purification of AuBPs

**Figure 1:**
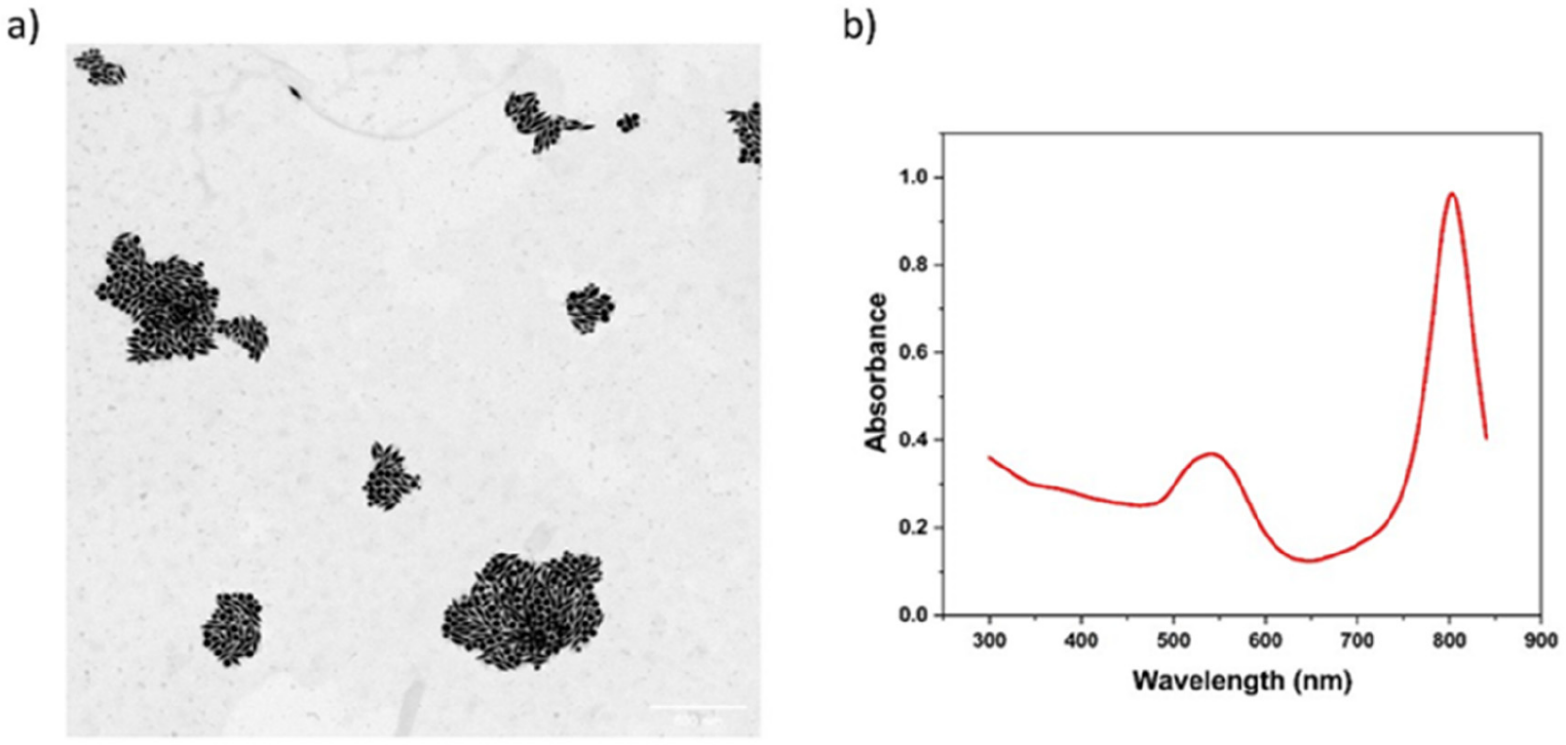
Characterization of as-synthesized AuBPs. (a) TEM image of AuBPs. The scale bar is 500 nm; (b) The UV–vis-NIR absorption spectrum of AuBPs.

The as-synthesized AuBPs possess a penta-twinned bipyramid structure with long and short axes of 87 × 31 nm, respectively, as shown in Figure 1a. The aspect ratio of the as-synthesized AuBPs was 2.8. Similar to previous studies, the as-synthesized products are composed of AuBPs and AuNPs.^24−26^ The corresponding UV–vis-NIR spectrum of the as-synthesized nanostructures is shown in Figure 1b, in which two characteristic absorbance peaks are observed at around 540 and 800 nm. This is consistent with previous studies, and the two absorbance peaks can be attributed to the longitudinal SPR (LSPR) and transverse SPR (TSPR).^27^ The spherical AuNPs contribute to the absorbance peak at about 540 nm, leading to a low ratio between LSPR and TSPR.^28^ By counting the nanoparticles recorded on TEM images (over 500 objects), it was estimated that the yield of AuBPs was only about 47% while the irregular-shaped nanostructures with different sizes accounted for the majority of the as-synthesized products. Different from the nanorods or nanocubes synthesized by the seed-mediated growth method which only uses NaBH_4_ as the reducing agent, the synthesis of seeds for AuBPs fabrication needs the addition of sodium citrate.^29−31^ In this situation, the citrate-stabilized seeds showed a multiply twinned crystals structure under TEM while the seeds for nanorods are single-crystalline.^31^ By conducting atomistic simulations of seeds during the growth process, it was found that the surfactant has a different coverage rate on the different facets of Au seeds, leading to a different growth rate of the nanoparticle in different directions and consequently the formation AuBPs.^32^ From this point of view, the low yield of AuBPs can be attributed to the defective crystal structure of Au seeds.^14^ To avoid aggregation and precipitation, the as-synthesized AuBPs were washed and redispersed in 5 mM CTAB solution. Through this, the AuBPs would be capped with CTAB molecules and exhibit excellent colloidal stability due to the electrostatic repulsion of the polarity head of CTAB.

**Figure 2:**
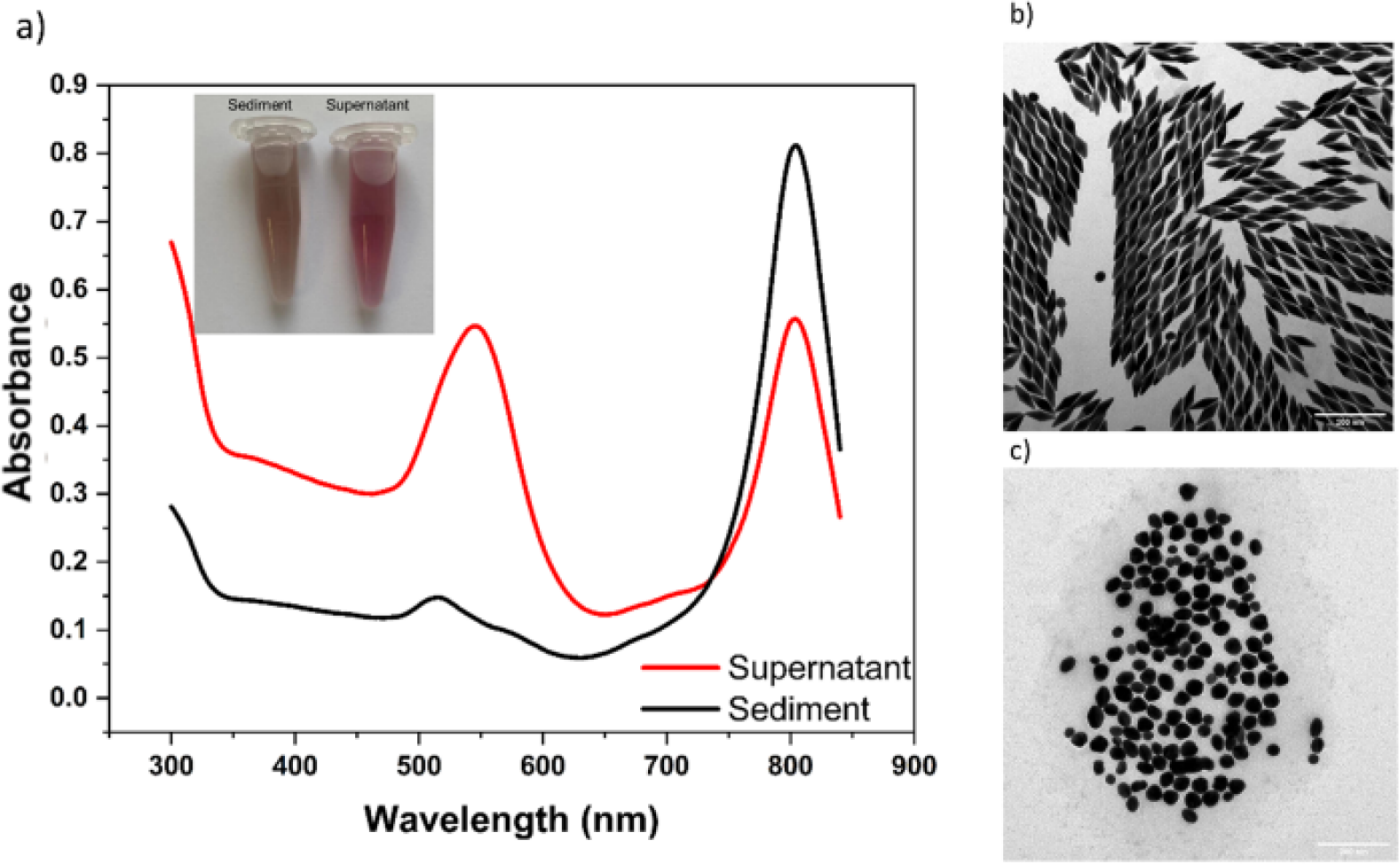
Purification of AuBPs. (a) UV–vis-NIR absorption spectra of unpurified AuBPs (red curve) and purified AuBPs (black curve). The inset shows the color of the purified AuBPs (sediment) and the supernatant; (b) TEM image of purified AuBPs; (c) TEM image of the supernatant containing irregular-shaped gold nanoparticles. The scale bars in (b) and (c) indicate 200 nm.

For purification of AuBPs, 6 mL of 4 M NaNO_3_ solution was added to 4 mL of the above AuBPs solution. The color of the solution changed immediately from red-violet to dark gray, indicating a quick aggregation of AuBPs. After the addition of salts solution, the mixture was heated up to around 50 °C, and once the color of the solution changed from gray to red, the mixture was placed in an incubator at different temperatures. After 12 h without disturbance, the red supernatant was carefully removed, and the pallet was redispersed in 10 mM CTAB solution by sonication. Subsequently, the redispersed AuBPs were washed twice to remove excess NaNO_3_ and dispersed in a 10 mM CTAC solution. As shown in the inset picture in Figure 2a, the color of the isolated AuBPs was brown, while that of the supernatant was red. In the UV–vis-NIR absorption spectrum in Figure 2a, the purified AuBPs have a much weaker absorbance at around 520 nm (Figure 2a, black curve) than the supernatant (Figure 2a, red curve). The intensity ratio between the LSPR at around 800 nm and TSPR at around 520 nm increased substantially from 2.6 to 5.5, indicating the increased ratio of AuBPs.^27^ Further experiments using this method to purify AuBPs of various sizes (Figures S3 and S4) demonstrated that the LSPR/TSPR ratio can be increased up to 6.7 for AuBPs with a size of long and short axes of 51.7 and 18.4 nm, respectively (Figure S5). This ratio between the LSPR and TSPR in this study is higher than that in previous studies (typically 4 to 5).^11,33−35^

Consistent with the finding from the UV–vis-NIR absorption spectrum, the TEM images in Figure 2b, S1a, show a very high purity of purified AuBPs. The yield of this batch of AuBPs reaches over 93% after a single round of purification. The yield is as high as 97% for AuBPs with a size of long and short axes of 51.7 and 18.4 nm, respectively, after a single round of purification. In contrast, the TEM images in Figures 2c and S2b illustrate that the supernatant is almost only composed of irregular-shaped nanoparticles. These results clearly demonstrated that we developed a facile and powerful purification process to separate AuBPs from other nanostructures with different shapes.

### 3.2 The Influence of Temperature and Electrolytes on Purification of AuBPs

**Figure 3:**
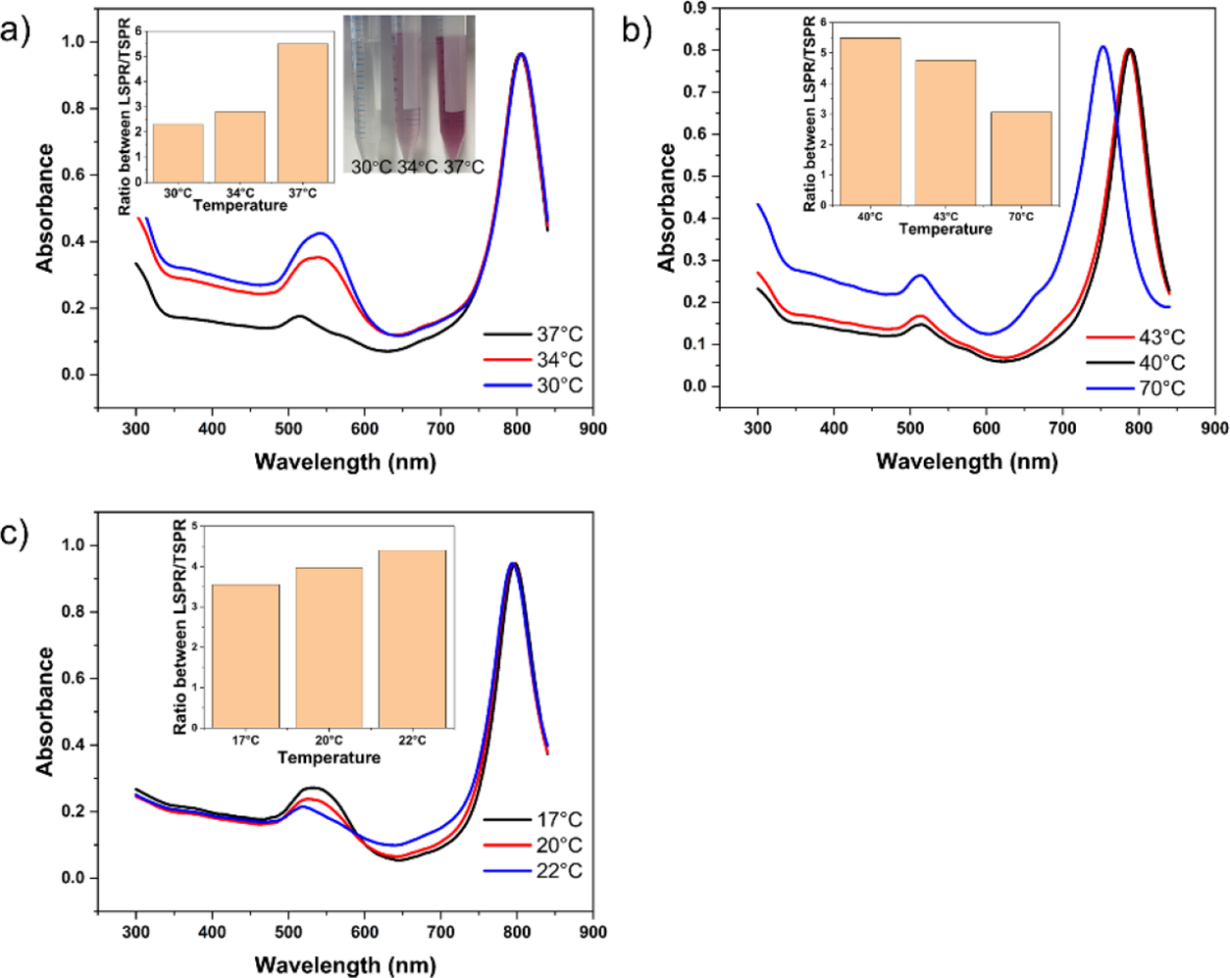
Isolation of AuBPs at different temperatures. (a) UV–vis-NIR spectrum of AuBPs purified by adding NaNO_3_ at 30 °C (blue curve), 34 °C (red curve), and 37 °C (black curve). The inset shows the purification samples incubated at 30 °C (left), 34 °C (middle), and 37 °C (right). (b) UV–vis-NIR spectra of AuBPs purified by adding NaNO_3_ at 40 °C (black curve), 43 °C (red curve), and 70 °C (blue curve). (c) The UV–vis-NIR spectra of AuBPs purified by adding NaCl at 17 °C (black curve), 20 °C (red curve), and 22 °C (blue curve). The inset histogram represents the ratio between LSPR/TSPR.

In order to investigate the influence of temperature on the purification of AuBPs, we set the incubation temperature to be 30, 34, and 37 °C. At 30 °C, the as-synthesized products were almost all aggregated and precipitated after 12 h (Figure 3a). The solution changed to colorless, and a pellet was observed at the bottom of the tube. It indicates that mixing the as-synthesized products and salts causes rapid aggregation of AuBPs and AuNPs at this temperature. The UV–vis-NIR spectrum of the redispersed pellet was the same as that of the sample before purification. The result suggests that AuBPs and AuNPs were all precipitated and no isolation occurred. When the temperature increased to 34 °C, the isolation was slightly better, demonstrated by marginally increased LSPR/TSPR ratio. By further increasing the incubation temperature to 37 °C, the color of solution changed from colorless to light pink to rose, indicating the increased retention of spherical nanoparticles in the supernatant. The UV–vis-NIR spectrum of the purified AuBPs revealed that the ratio between the LSPR and TSPR increases with incubation temperature, peaking at 5.49 at 37 °C. This suggests that, in the range of 30 to 37 °C, the AuBPs were not stable and intended to precipitate while the colloidal stability of AuNPs enhanced with increasing temperature. Therefore, by increasing the temperature, AuNPs were able to be removed from the solution, and the purification was improved with increased temperature within the temperature range of 30 to 37 °C.

In order to further investigate the purification efficiency at higher temperature, we continued to increase the temperature to 40, 43, and 70 °C. As shown in Figure 3b, the highest ratio between the LSPR and TSPR was achieved at 40 °C, peaking at 5.51, indicating the best purification results. If the isolation temperature further increased to 43 and 70 °C followed by incubation of 12 h, a decrease in the ratio between LSPR and TSPR was observed. Besides, with the temperature increase, the peak showed a blue-shift. According to the previous study, at high temperature, the atoms at the tips of AuBPs tend to undergo structural rearrangements to minimize surface energy, leading to shape changes and poor thermal stability.^36^ Therefore, 37 °C was selected as the best isolation temperature for using NaNO_3_ to isolate AuBPs for achieving both high purity and high quality.

To test whether other electrolytes can also be used in the purification of AuBPs, we used NaCl in the following experiments. While previous studies utilized NaCl in the purification of nanostructures, the influence of temperature was not explored.^37,38^ We added a 4 M NaCl solution to the AuBPs solution and then incubated the mixture at 17, 20, and 22 °C. As shown in Figure 3c, the purification of AuBPs by NaCl was also confirmed to be influenced by temperature. At a low temperature of 17 °C, similar to the results of purification by NaNO_3_, the solution turned colorless after incubation for 12 h, indicating that almost all nanostructures were aggregated and precipitated. In this situation, a very limited purification result was achieved. By increasing the temperature, the purification efficiency improved. At 22 °C the highest ratio between the LSPR and TSPR was recorded at 4.4. However, when a higher temperature of over 22 °C was applied, no precipitation could be observed after 12 h. It indicated that both AuBPs and AuNPs were stable at high temperatures. In conclusion, for purification of AuBPs by NaCl, at a temperature below 17 °C, no obvious isolation was achieved; at a temperature between 17 and 22 °C, the higher the isolation temperature, the better the purification result; at a temperature of 23 °C or above, both AuBPs and AuNPs were stable; therefore, the isolation could not be observed. It is apparent that temperature plays a significant role in purification of AuBPs. Therefore, it is of great importance to study and recognize the influence of temperature on the purification of AuBPs, particularly when different electrolytes or salts are used.

### 3.3 Mechanism of AuBPs Separation

In solution, the quaternary ammonium headgroup of CTAB molecules binds to gold nanostructures and CTAB molecules form a cationic surfactant bilayer to maintain colloidal stability.^39^ The absorption of CTAB molecules on gold nanostructures is a dynamic process, and there is a rapid dynamic equilibrium between the CTAB molecules on the nanostructures and the free CTAB in the solution.^40^ It has been believed that the stability of this kind of colloidal system will be affected by the addition of salts due to the influence on the electrostatic interaction between nanostructures. To investigate whether the stability of different gold nanostructures and the consequent separation is mainly caused by the change in electrostatic interaction induced by electrolytes, we added two different types of salts with the same amount and concentration to our as-synthesized product for AuBPs separation. First, we added 3 mL of 0.1 M NaSal to 3 mL of AuBPs solution and then kept the tube individually at 18 and 37 °C for 12 h. As shown in Figure S6, the isolation results are similar to those using NaNO_3_ and NaCl: the higher the incubation temperature, the better the purification result. However, when we added 3 mL of 0.1 M NaCl to the AuBPs solution, no sediment was observed. These results suggest that the ionic strength and electrolyte concentration were not the key factors that determine the stability of nanostructures and consequently the purification results; and there should be other factors which were influencing the colloidal stability in the separation process.^11,37^

A different opinion believes that the free CTAB micelles play an important role in stabilizing nanostructures. Skoglund et al. proposed an explanation that silver NPs are stabilized by the cluster of the surfactant micelles in the vicinity of the NPs rather than the absorbed CTAB molecules on the NPs.^41^ Similarly, Schmutzler et al. proposed that the stability of AuNPs is not dependent on the CTAB bilayer on the NPs but the morphology of CTAB micelles.^42^ Based on these explanations, in this study, we proposed that the change of the structure of CTAB micelles under the action of electrolyte played an important role in the temperature-derived purification.

**Figure 4:**
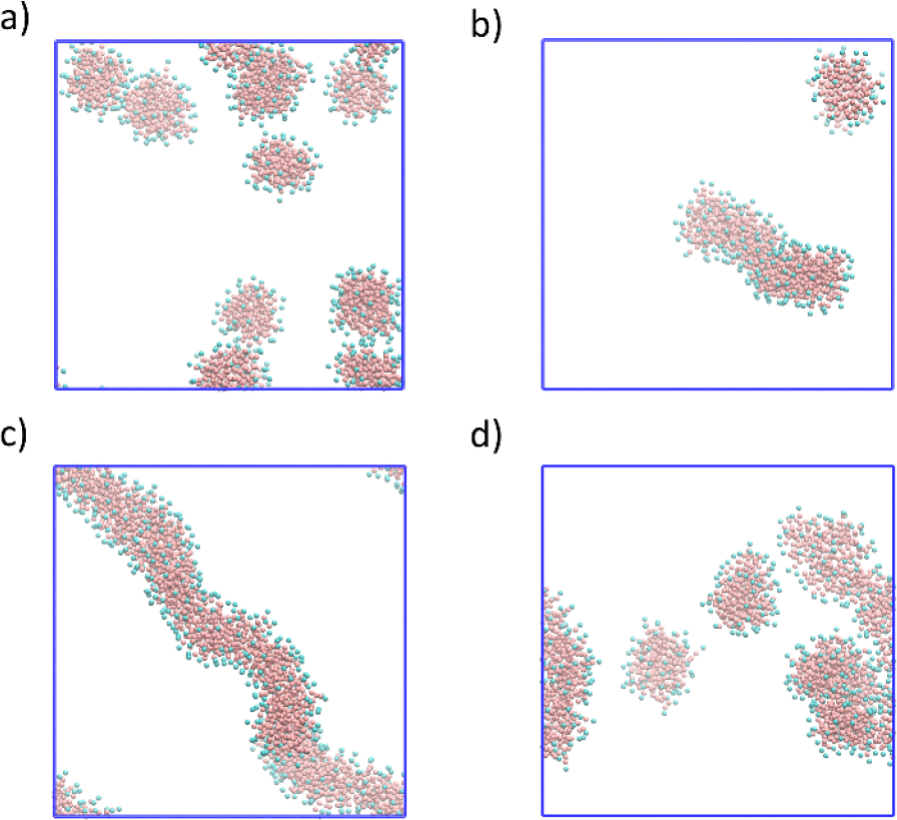
Representative snapshots for CTAB micelles at different conditions: (a) 0.1 M CTAB molecules solvated in 0.1 M NaCl solution at 15 °C; (b) 0.1 M CTAB molecules solvated in 0.05 M NaSal solution at 15 °C; (c) 0.1 M CTAB molecules solvated in 0.1 M NaSal solution at 15 °C; (d) 0.1 M CTAB molecules solvated in 0.05 M NaSal solution at 80 °C. The hydrophilic ammonium heads of CTAB molecules are represented as light blue sphere, while the hydrophobic tails are shown as pink sphere. The water and salt particles are not shown for clarity.

It has been reported that, when salts, such as NaCl, NaNO_3_, and NaSal, are added to CTAB solution, the CTAB micelles may undergo a spherical-to-wormlike micelle transition.^43,44^ To further investigate how the temperature influenced the structure of CTAB micelles, we performed CG MD simulations to explore the influences of the salt type and salt concentration, as well as temperature, on the shape of CTAB micelles. Our simulation results indicate that the type of salts, salt concentration, and temperature exert significant influence on the shape feature of CTAB micelles. As shown in Figure 4, CTAB molecules tend to aggregate to form long stable worm-like micelles in NaSal solutions, while they will form short sphere-like micelles in NaCl solutions at a low temperature of 15 °C. This phenomenon is consistent with previous work, which has proven that the hydrophobic NaSal salt has more pronounced effects on the formation of micelles compared with simple inorganic NaCl.^45,46^ The difference in CTAB micelle shape between Figures 4b and 4c shows that the increase of salt concentration increases the micellar length. The increase of Sal^–^ concentration enhances the effects of Sal^–^ to shield the repulsive interactions among the heads of CTA^+^, which promotes the elongation of CTAB micelles.^47,48^ By heating the solutions from 15 to 80 °C, the worm-like CTAB micelles become unstable, and the micelles break into smaller pieces. As a result, the length of micelles is shortened and the number of micelles is increased. In a previous study, Sambasivam et al. presented a perspective that the wormlike and rodlike CTAB micelles can associate them with nanoparticles and from micelle-nanoparticle aggregates.^49^ Here, we speculated that the interactions with rodlike and wormlike micelles caused the aggregation of gold nanostructures. With the addition of salt, the wormlike CTAB micelles appear, resulting in the aggregation of nanostructures and the color change. When the temperature changes, the shape of CTAB micelles changes as well. Overall, we believe that the stability of gold nanostructures and the consequent separation of different shapes of gold nanostructures are determined by several factors: the concentration and type of salts, the temperature of the solution, and the shape and size of the nanostructures themselves.

In our study, when we incubated the gold nanostructure solution at high temperatures, the CTAB micelles tended to transit to spherical or shorter rodlike micelles. In this situation, the interaction between CTAB micelles and gold nanostructures kept them stable. The type of electrolytes also influenced the shape of CTAB micelles. For example, at the same concentrations, NaSal is preferable to facilitate the spherical-to-wormlike micelle transition compared to NaCl. Therefore, by adding 0.1 M NaSal, the aggregation of AuBPs was observed, while they were kept stable when the same concentration of NaCl was added. Besides the influence of surfactant micelles, the stability of nanoparticles is also subjected to other factors, including size, shape, viscosity, and ionic strength. In comparison with small spherical AuNPs, AuBPs possess larger dimension and larger surface, and therefore, AuBPs were susceptible to sedimentation first when the colloidal stability decreased.^11,50^ By alteration of the temperature, the colloidal stability can be adjusted. At a specific temperature, large AuBPs sediment, while small spherical nanoparticles keep stable in the solution, and thus purification of AuBPs can be achieved. Overall, we speculate that the difference in the optimum temperature for purification of AuBPs by different salts is associated with their different abilities in triggering the structural transition of CTAB micelles. Because of the different physicochemical properties of counterions, the size and shape of CTAB micelles vary with addition of different salts and the incubation temperature, which consequently influences the colloidal stability of nanostructures. In order to purify AuBPs, the salt concentration and purification temperature can be carefully adjusted to ensure that small spherical particles remain stable in the supernatant while large AuBPs precipitate. Therefore, in the purification of AuBPs, the optimal concentrations and temperatures are different for different salts.

However, it should be noted that the aggregation of nanostructures in a salt solution is a complex process. The introduction of electrolytes can shield the electrostatic repulsion and change the colloidal stability. On one hand, when the temperature increases, the wormlike micelles shorten and viscosity decreases.^45^ On the other hand, the Brown motion of nanostructures increases with temperature. Besides, the depletion force also plays an important role in the sedimentation of AuBPs. When the distance between nanoparticles is small enough, depletants are forced out from the depleted zone, creating an osmotic pressure that pushes the nanoparticles together.^51,52^ The depletion force is affected by many factors, and one of them is the topology of the nanoparticles.^53,54^ The depletion free energy change is proportional to the overlap volume of depletion zones.^54,55^ For a fixed particle volume, spherical nanoparticles have the least overlap volume of depletion zones.^53^ Therefore, large AuBPs are subjected to more depletion force and tend to aggregate, while the small AuNPs are subjected to less depletion force and are more stable in the solution. Therefore, the temperature-derived isolation of nanostructures should be balanced by multiple factors. There is still a need to understand how these factors influence colloidal stability as well as the isolation of nanostructures.

### 3.4 Detection of TA

Following the successful purification of AuBPs, we developed a colorimetric method to apply them to detect TA. TA is a natural polyphenol with a central core of glucose that is esterified with five digallic units that can be secreted by plants.^56^ The commercial TA is usually obtained from twigs of trees, such as chestnut and oak trees. TA is widely used in the industry.^57^ However, TA is regarded as moderately toxic by inhalation and ingestion and may cause environmental pollution. Therefore, it is of great use to develop a simple and sensitive method to detect TA. For the colorimetric detection of TA, a certain amount of AgNO_3_ was added to the AuBP solution followed by the addition of TA. Structurally, TA contains multiple phenol groups and hydroxyl groups. These phenol groups and hydroxyl groups possess the reducibility which can reduce the silver ion to the silver atom at specific pHs. The reduced silver atom can then grow on the surface of AuBPs and result in a blue shift of the SPR peak. Correspondingly, measuring the change of the SPR peak is able to quantify the amount of TA. In order to achieve the best performance of colorimetric detection, the amount of AuBPs and reaction time were first optimized. We examined the influence of AuBPs on the sensitivity of the detection by adding different amounts of AuBPs (600, 750, 900, 1050, and 1200 μL) with 100 μL of TA. The result reveals that the amount of AuBPs plays an important role in the detection of TA. As shown in Figure S7, with the increase of the amount of AuBPs the shift of SPR peak (Δλ) decreases. The reason is that, with less AuBPs, the reduced silver atom can form a thicker shell on each AuBP, leading to a great change in Δλ. On the whole, with the reduced amount of AuBPs, the sensitivity is better but the detection range is smaller. Therefore, in order to achieve relatively high sensitivity and a relatively wide detection range, 900 μL of AuBPs was used in the experiments. Furthermore, we optimized the reaction time for the detection of TA (Figure S7). By heating the mixture at 60 °C, the color of the solution changed gradually. In the initial 10 min, a remarkable shift of the SPR peak was observed. From 10 to 70 min, the change of SPR peak slowed down and reached steady-state after incubation for 70 min. Hence, the time point of 70 min was set as the reaction time in detection.

**Figure 5:**
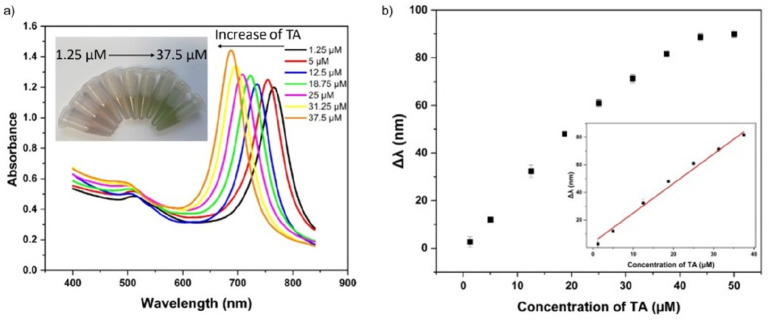
(a) Image and corresponding UV–vis-NIR spectrum of AuBPs for the colorimetric detection of TA; (b) The linear relationship between the change of SPR absorption of AuBPs and TA concentration.

Figure 5a shows the color change of AuBPs in detecting different amounts of TA. When the concentration of TA in the sample increases from 1.25 to 37.5 μM, the color changes gradually from brown to green. Correspondingly, the SPR peak blue shifts from 766 to 686 nm. As shown in Figure 5b, a good linear relationship is observed between the concentrations of TA and Δλ.

In order to understand the changes of the AuBPs morphology during detection and the relationship between the nanoparticle shape and TA concentration, the TEM images of AuBPs were obtained. As shown in Figure S8, the AuBPs have undergone a shape change after incubation with TA and AgNO_3_. The shape changed from pyramid-shaped to spindle shape nanoparticles. Consistent with the previous study, when the Ag^+^ ions are reduced by the reductant in the system, the reduced Ag atoms deposit on AuBPs and grow along the long axis.^58^ According to Mie theory, the scattering of electromagnetic waves on the surface of nanostructures is highly related to the shape, size, and the material of the nanoparticle.^59^ Therefore, when the growth of silver on AuBPs happens, the dielectric constant, shape, and size of the nanostructures change, resulting in shift of the SPR peak. At the optimized conditions, the calibration equation for the detection of TA was defined as Δλ = 3.705 + 2.1386*C*_TA_ with *R*^2^ = 0.9858. The limit of detection (LOD) was calculated as 0.86 μM with a demonstrated detection range from 1.25 to 37.5 μM.

### 3.5 Selectivity for TA Sensing

In order to evaluate the selectivity of the method for TA detection, several interference chemicals were analyzed, including NaNO_3_, CaCl_2_, NaCl, glucose, sodium acetate, glycolic acid, glutamine, quinic acid, and urea. Samples containing 20 mM of these inference chemicals were added to the AuBPs solution using 25 μM TA as a control group for comparison. As shown in Figure S10, after the addition of interference chemicals, no obvious SPR peak shift was observed, and the color of the solution remained the same. On the contrary, when TA was added to the AuBPs solution, a significant color change was recognized by naked eyes, accompanied with significant SPR peak shift of 67 nm. Considering that the concentration of the interference chemicals was 800 times higher than that of TA, the results demonstrated the good selectivity of this approach for TA detection. It should be noted that Ag^+^ may react with Cl^–^ to form insoluble AgCl. However, in our study, no significant interference was observed when chloride salts were added. One possible reason is that, in our study, the concentrations of Ag^+^ and Cl^–^ were relatively low; hence, there was no apparent precipitation.

### 3.6 Real Sample Analysis

To demonstrate the practicability of the developed colorimetric method for the detection of TA, we investigated the accuracy of the method for TA detection in tap water. Different amounts of TA were added to the tap water samples, and then the concentration was quantified by the developed colorimetric method. As shown in Table 1, the detected values of the TA concentration were in the range of 101.46–105.9% of the real concentrations, with an RSD of less than 6.8%. The slightly higher recovery rate may be attributed to the higher pH value of tap water than water. The results demonstrated the potential of using our developed approach for the detection of TA in real samples.

**Table 1:** Colorimetric Detection of TA in Spiked Water Samples

Sample	Known Concentration (μM)	Detected Concentration (μM)	Recovery Rate (%)	RSD (%, *n* = 3)
Sample 1	27.5	27.9	101.5	0.3
Sample 2	16.5	17.3	104.8	4.0
Sample 3	9.5	10.1	106.3	6.8

## 4 Conclusion

In summary, we developed a simple but powerful method to isolate and purify AuBPs. By adding cheap and easily available salts and adjusting the temperature, AuBPs can be purified with purity and LSPR/TSPR ratios as high as 97% and 6.7, respectively. Our approach is highly reproducible by considering the significant influence of temperature on purification of AuBPs. Furthermore, our study investigated the mechanisms of this phenomenon. Through CG MD simulation, we explored that the shape of CTAB micelles changes with the temperature of the solution, the concentration, and types of salts. Under different conditions, the CTAB micelles can be either spherical or wormlike. By controlling the parameters, a spherical-to-wormlike micelle transition can be observed and made use for purification of AuBPs, as the CTAB micelles play an important role in supporting the stabilized dispersion of gold nanostructures. It is expected that this facile approach will be extended to the purification of many different types of nanostructures. With the purified AuBPs, we developed a colorimetric approach for selective and sensitive detection of TA. The color change of AuBPs solution with various concentrations of TA can be easily distinguished by naked eyes. A linear range of 1.25 to 37.5 μM and an LOD of 0.86 μM were achieved.

## Supporting Information Available

TEM images, UV-vis-NIR spectra, optimization of experiment parameters, wavelength shift, comparison of reported methods for TA detection, additional refs (PDF)
