# Supplementary material for: Temperature-Derived Purification of Gold Nano-Bipyramids for Colorimetric Detection of Tannic Acid
Source: ACS Appl Nano Mater. 2023 Jun 27;6(13):11572–80. doi: 10.1021/acsanm.3c01593 (PMC10353004; doi:10.1021/acsanm.3c01593)
Supplement: Supplementary file 1 — an3c01593_si_001.pdf [file an3c01593_si_001.pdf]

Supporting Information

Temperature-Derived Purification of Gold Nano-Bipyramids for Colorimetric Detection of Tannic Acid

Yuxiang Xue,† Xinyao Ma,‡ Xue Feng,† Sam Roberts,† Guangyu Zhu,¶ Yi Huang,§ Xianfeng Fan,§ Jun Fan, ‡ and Xianfeng Chen†**

†School of Engineering, Institute for Bioengineering, University of Edinburgh, The King's Buildings, EH9 3JL, Edinburgh, UK

‡Department of Materials Science and Engineering, City University of Hong Kong, 83 Tat Chee Ave, Kowloon Tong, Hong Kong, SAR (P. R. China

¶Department of Chemistry, City University of Hong Kong, 83 Tat Chee Ave, Kowloon Tong, Hong Kong, SAR (P. R. China

§School of Engineering, Institute for Materials Processing, University of Edinburgh, The King's Buildings, EH9 3JL, Edinburgh, UK

*Corresponding authors. E-mails: junfan@cityu.edu.hk (Jun Fan); Michael.Chen@ed.ac.uk (Xianfeng Chen)

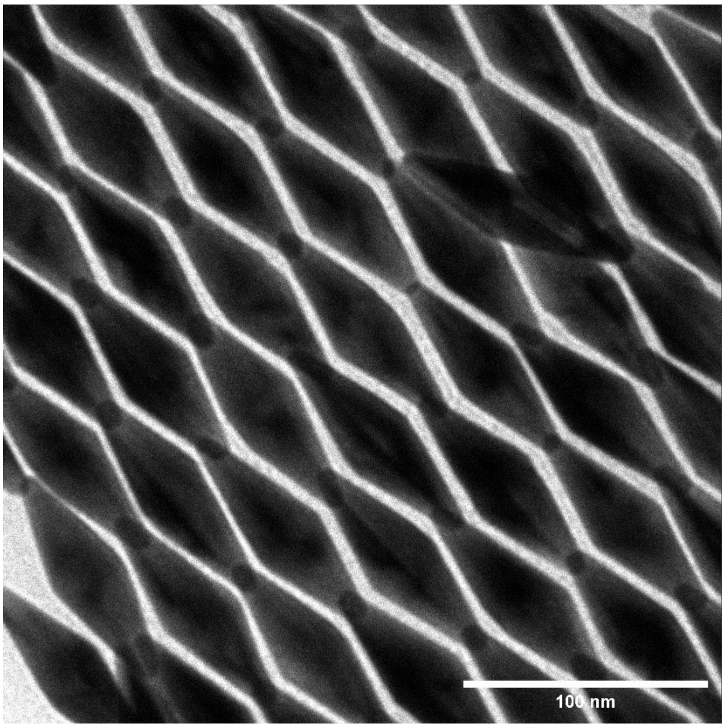

Figure S1. TEM image of the morphology of prepared AuBPs. The scale bar is 100 nm.

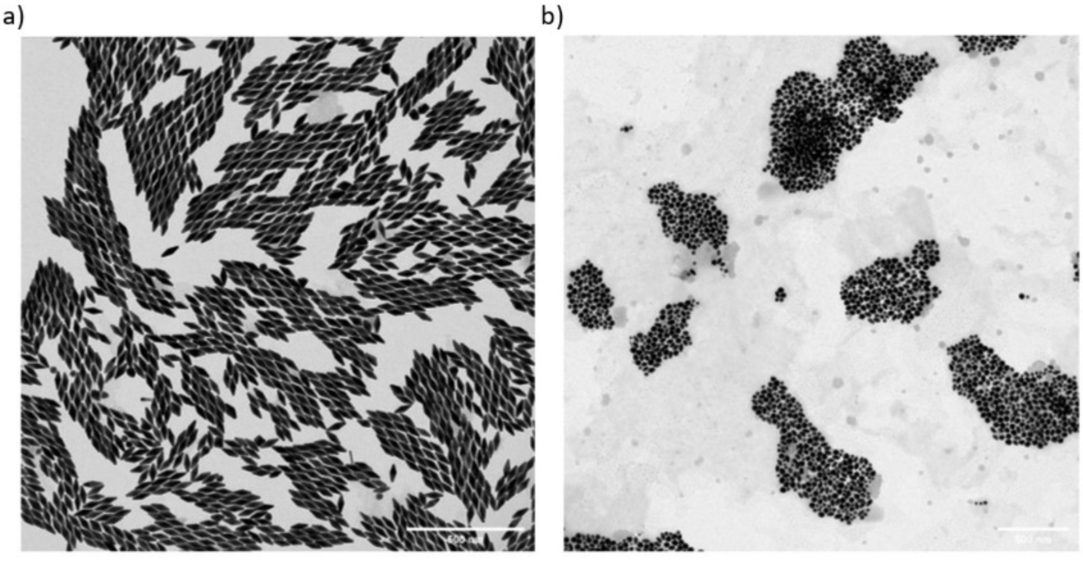

Figure S2. TEM image of (a) purified AuBPs and (b) supernatant. The scale bars are 500 nm.

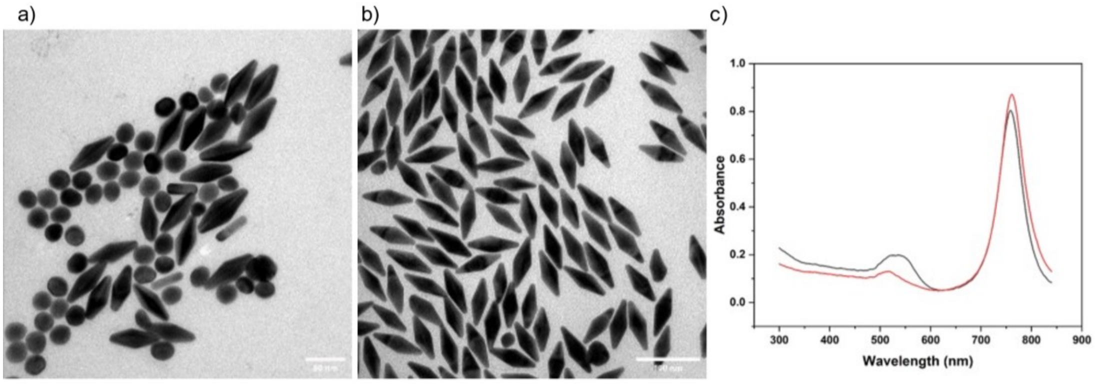

Figure S3. TEM characterisation of purification of AuBPs with a size of long and short axes of 70.5 × 23.6 nm, respectively. (a) TEM image of as synthesised AuBPs; (b) TEM image of AuBPs after purification. The scale bars in a) and b) indicate 100 nm. (c) UV-Vis-NIR spectra of as-synthesised AuBP (black curve) and purified AuBPs (red curve).

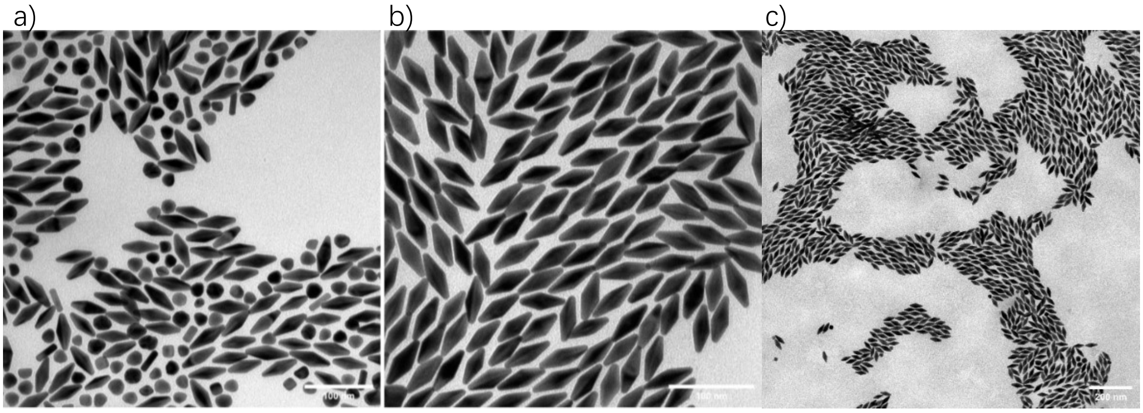

Figure S4. TEM characterisation of purification of AuBPs with a size of long and short axes of 51.7 × 18.4 nm, respectively. (a) TEM image of as synthesised AuBPs; (b) and (c) TEM images of AuBPs after purification at different magnifications. The scale bars in a) and b) indicate 100 nm. The scale bar in c) indicates 200 nm.

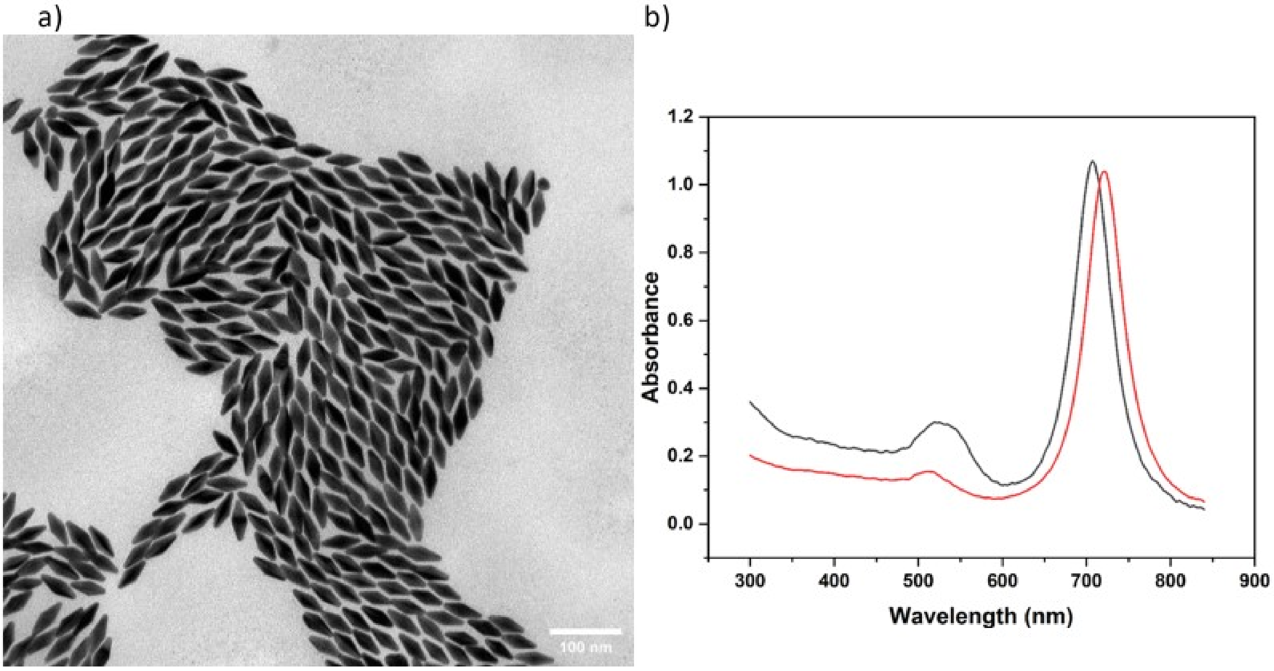

Figure S5. (a) TEM image of purified AuBPs with a size of long and short axes of 51.7 × 18.4 nm, respectively. The scale bar indicates 100 nm. (b) UV-Vis-NIR spectra of as-synthesised AuBP (black curve) and purified AuBPs (red curve). The ratio between LSPR/TSPR of the purified AuBPs is 6.7.

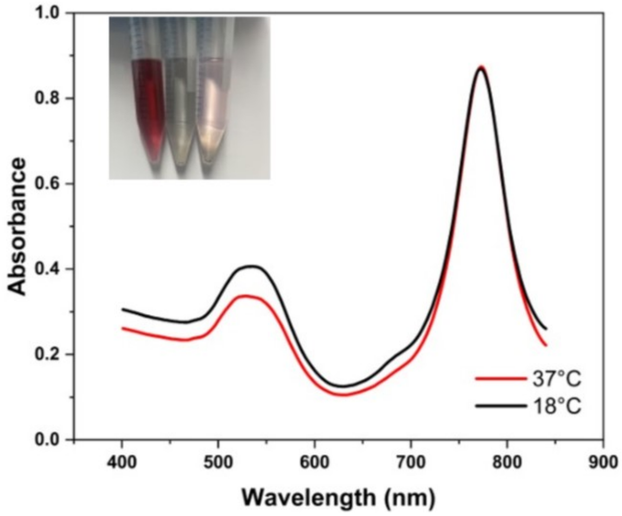

Figure S6. The UV-Vis-NIR spectra of AuBPs purified at 37 ºC (red curve) and 18 ºC (black curve). The inset photograph shows the result of AuBPs incubated with 0.1 M NaCl at 18 ºC (left) and with NaSal at 18 ºC (middle) and 37 ºC (right). The results indicate that the purification will be simultaneously influenced by temperature and type of salts.

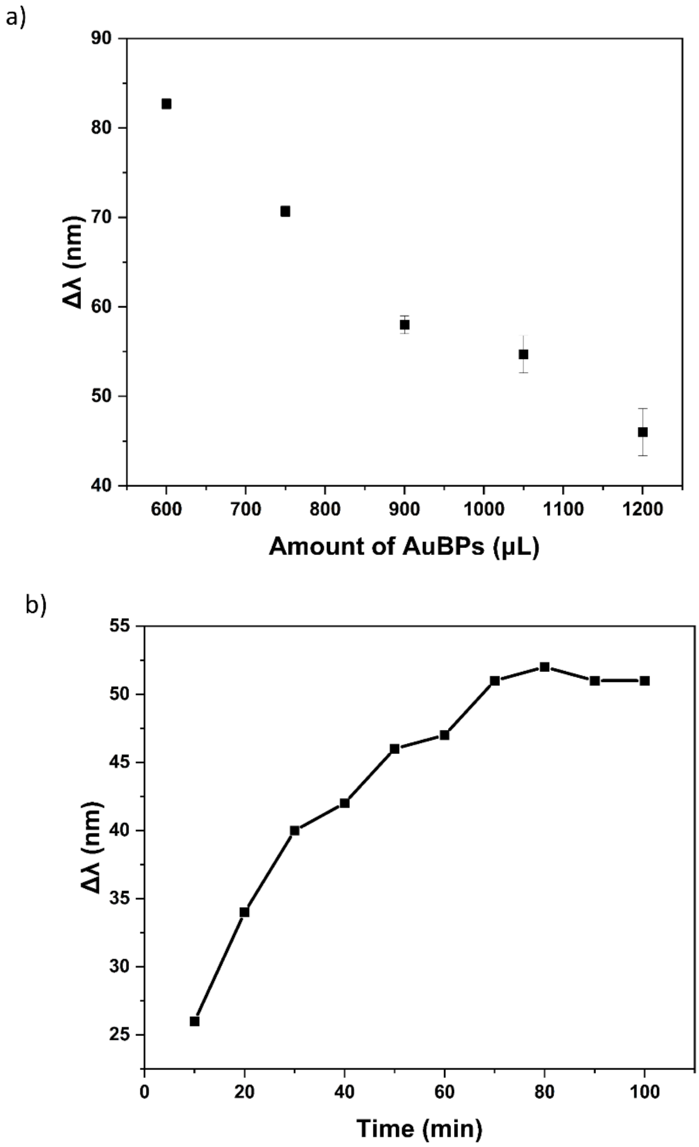

Figure S7. Optimisation of the experiment parameters in detection of TA. (a) Wavelength shift of SPR peak at different amounts of AuBPs; (b) The effect of incubation time on the shift of SPR peak in detection of TA.

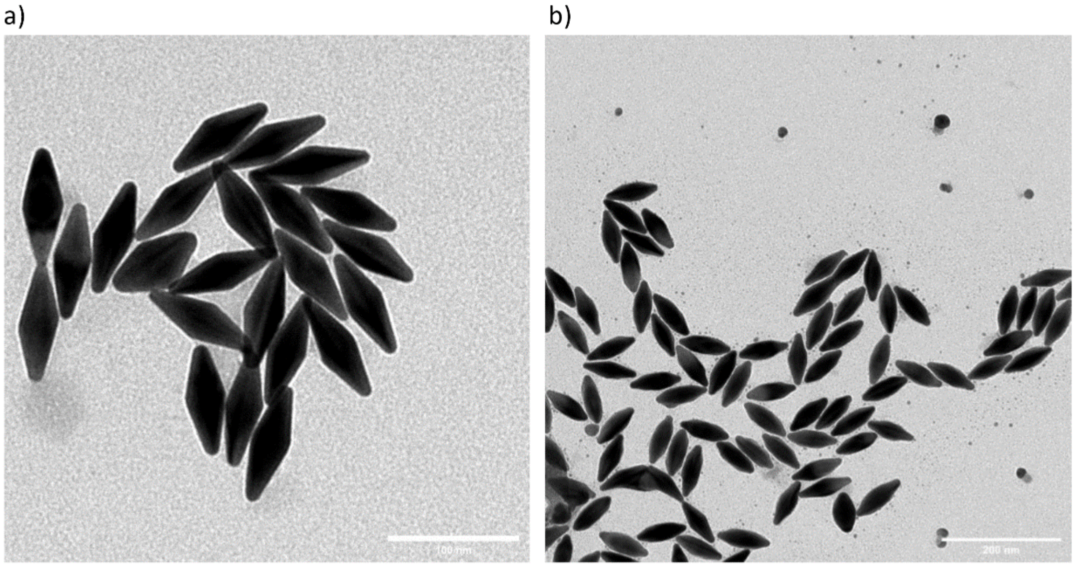

Figure S8. TEM characterisation of AuBPs after incubation with (a) 9.375 and (b) 37.5 μM TA sample. The scale bars in a) and b) indicate 100 nm and 200 nm, respectively.

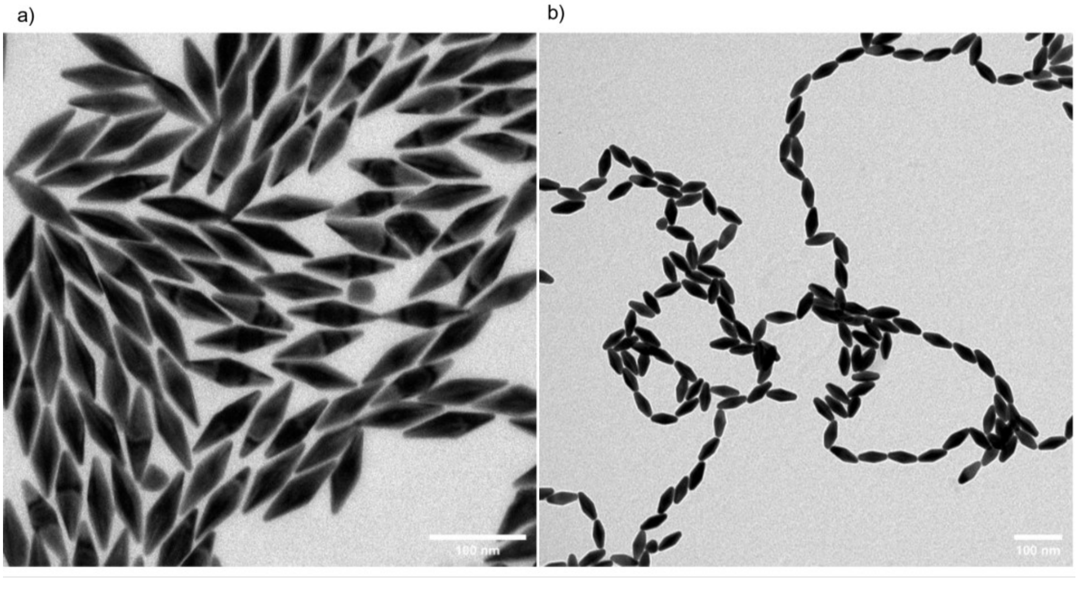

Figure S9. (a) TEM image of freshly prepared AuBPs; (b) TEM image of AuBPs after incubation in an incubator at 70 °C for 10 days. The scale bars in a) and b) indicate 100 nm.

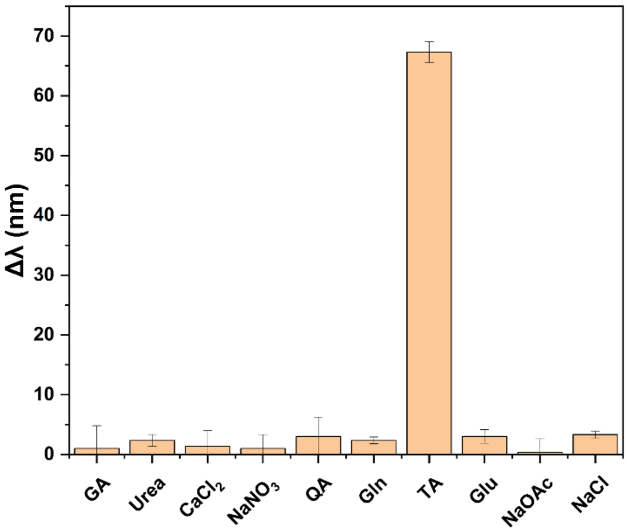

Figure S10. SPR shift of AuBPs in the presence of $AgNO_3$ and different chemicals.

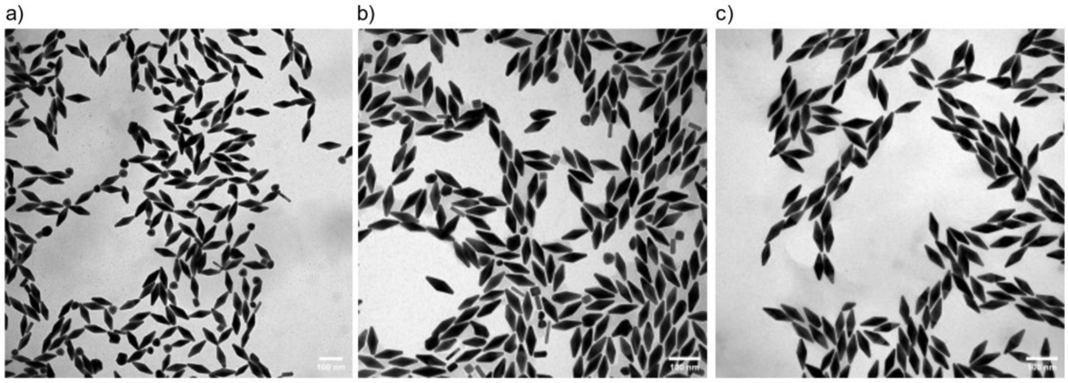

Figure S11. TEM image of purified AuBPs by adding $NaNO_3$ at (a) 30 ºC, (b) 34 ºC, and (c) 37 ºC. The scale bars in a), b) and c) indicate 100 nm.

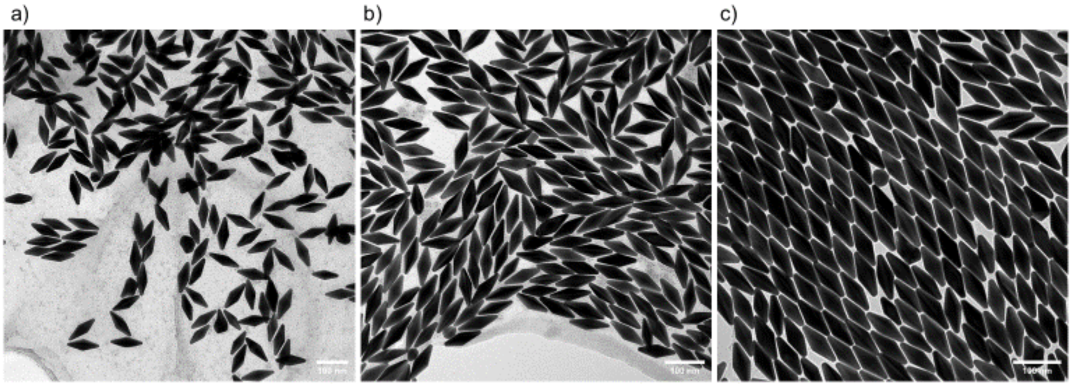

Figure S12. TEM image of purified AuBPs by adding NaCl at (a) 17 ºC, (b) 20 ºC, and (c) 22 ºC. The scale bars in a), b) and c) indicate 100 nm.

Table S1. Comparison of the performance of our method with different reported methods for TA detection.

Methods	Linear Range (μM)	Detection Limit (μM)	Reference
Colorimetric detection	1-100	1	1
Fluorescence analysis	0.4-9	0.12	2
Flow injection	40-147	10	3
Electrochemical analysis	2-42	0.6	4
Different pulse voltammetry	10-170	1	5
Colorimetric detection	1.25-37.5	0.86	This work

Reference

(1) Chen, Z.; Zhang, X.; Cao, H.; Huang, Y. Chitosan-Capped Silver Nanoparticles as a Highly Selective Colorimetric Probe for Visual Detection of Aromatic Ortho-Trihydroxy Phenols. *Analyst* **2013**, *138* (8), 2343–2349.

(2) Yang, H.; He, L.; Pan, S.; Liu, H.; Hu, X. Nitrogen-Doped Fluorescent Carbon Dots for Highly Sensitive and Selective Detection of Tannic Acid. *Spectrochim. Acta Part A Mol. Biomol. Spectrosc.* **2019**, *210*, 111–119.

(3) Cheng, T.-J.; Hsiao, H.-Y.; Chung, C.-Y.; Chen, P.-C.; Chen, R. L. C. Determination of Tannic Acid after Precipitation with Bovine Serum Albumin by Visible Light Scattering in a Flow Injection System. *Microchim. Acta* **2010**, *169*, 117–122.

(4) Yılmaz, Ü. T.; Çalık, E.; Uzun, D.; Karipcin, F.; Yılmaz, H. Selective and Sensitive Determination of Tannic Acid Using a 1-Benzoyl-3-(Pyrrolidine) Thiourea Film Modified Glassy Carbon Electrode. *J. Electroanal. Chem.* **2016**, *776*, 1–8.

(5) Tsai, T.; Yeh, P.; Chen, S.; Ali, M. A.; Al-Hemaid, F. M. A. Effect of Electrostatic Interaction on Electrodeposition of Nickel Hexacyanoferrate with Functional MWCNTs and Their

Application for the Determination of Persulfate and Tannic Acid. *Electroanalysis* **2014**, *26* (5), 971–979.
